# LC-MS/MS assisted biomonitoring of ropivacaine and 3-OH-ropivacaine after plane block anesthesia for cardiac device implantation

**DOI:** 10.3389/fmolb.2023.1243103

**Published:** 2023-09-27

**Authors:** Mihaela Butiulca, Lenard Farczadi, Camil Eugen Vari, Silvia Imre, Mihai Pui, Alexandra Lazar

**Affiliations:** ^1^ Department of Anesthesiology and Intensive Care Medicine, Faculty of General Medicine, George Emil Palade University of Medicine, Pharmacy, Science, and Technology, Târgu Mureș, Romania; ^2^ Department of Anesthesiology and Intensive Care Medicine, Emergency County Hospital, Târgu Mureș, Romania; ^3^ Chromatography and Mass Spectrometry Laboratory, Center for Advanced Medical and Pharmaceutical Research, George Emil Palade University of Medicine, Pharmacy, Science, and Technology, Târgu Mureș, Romania; ^4^ Department of Pharmacology and Clinical Pharmacy, Faculty of Pharmacy, George Emil Palade University of Medicine, Pharmacy, Science, and Technology, Târgu Mureș, Romania; ^5^ Department of Analytical Chemistry and Drug Analysis, Faculty of Pharmacy, George Emil Palade University of Medicine, Pharmacy, Science, and Technology, Târgu Mureș, Romania

**Keywords:** ropivacaine, metabolite, LC-MS, biomonitoring, anesthesia

## Abstract

**Introduction:** Ropivacaine is a popular local anesthetic used for regional anesthesia or for pain management. Although designed as an enantiomerically pure drug, an aspect that reduces the adverse effects, its toxicological effects are still a risk. As such, biomonitoring to assure appropriate dosage and bioavailability are essential to avoid complications during or post-surgery.

**Methods:** The study focused on developing a sensitive, selective, and accurate liquid chromatography—mass spectrometry (LCMS/MS) method which facilitates the biomonitoring of ropivacaine and its main metabolite in plasma after regional anesthesia using ropivacaine.

**Results and Discussion:** The method was validated with regards to all relevant parameters, such as sensitivity, selectivity, accuracy, precision, and the effect of sample matrix. The method was successfully used in a pilot study, which included one patient undergoing plane block anesthesia for cardiac device implantation. The results showed the method is appropriate for its intended purpose and could even be used in other, similar applications.

## 1 Introduction

Ropivacaine is the first long-acting enantiomerically pure local anesthetic used for regional anesthesia. It has a similar structure as bupivacaine and mepivacaine (Dossou et al., 2011). It is a pure S (−) enantiomer, developed as a safer anesthetic, for the purpose of reducing cardiac and neurologic toxicity (Hansen, 2004). Enantiomers exist in two spatial configurations, present in equal amounts in a racemic mixture. Even though they are similar from a physicochemical standpoint, the two enantiomers behave differently regarding their affinity to action sites. The difference consists of a greater affinity to the desirable action site *versus* affinity to action sites involved in generating side effects. The S (−) enantiomer has been proven to have fewer adverse effects compared with the R (+) enantiomer (Aberg, 1972).

### 1.1 Clinical uses

Ropivacaine is mainly used for surgical anesthesia, labor pain, and management of postoperative pain in adults and children. It can be administrated epidurally (usually for cesarian section and hip or lower limb surgery, but can also be used following abdominal surgery), intrathecally, or peripheral nerve blocks can be performed either for anesthesia or for management of pain post-surgery. Ropivacaine can also be beneficial in patients with chronic pain, or malignant pain. It has been proven that it is more effective in relieving pain when used as a local analgetic than intravenous morphine, while with a lower risk of complications than general anesthesia (Graff et al., 2021).

### 1.2 Mechanism of action

Regarding the mechanism of action, ropivacaine diminishes sodium ion influx through the nerve cellular membrane, reversibly blocking the conduction of the nerve impulse. The blockade of impulse conduction in nerve fibers is caused by the reversible suppression of sodium ion inflow (Hansen, 2004). Dose-dependent inhibition of potassium ion channels is also present with an additive effect (Kindler et al., 2003). The lower lipophilicity of ropivacaine compared with bupivacaine explains its selective action on pain transition (A, and C nerves), rather than large, myelinated motor fibers (A fibers) (Graf et al., 2002a).

### 1.3 Chemical structure

Ropivacaine is a part of pipecoloxylides group. It has a propyl group on the piperidine nitrogen atom, compared to butyl group present in bupivacaine (Ropivacaine, 1995). By replacing the methyl group (-CH_3_) linked to the nitrogen atom with a propyl group (-C_3_H_7_) in the case of ropivacaine and a butyl group (-C_4_H_9_) in the case of bupivacaine, both these substances are related to mepivacaine, since the carbon side chain connected to the tertiary nitrogen atom is the only difference between ropivacaine, bupivacaine, and mepivacaine. The lipophilicity also varies with carbon chain length, which has an impact on the potency of the different local anesthetics. The presence of an asymmetric carbon conducts to R (−) and S (−) enantiomers, altering the biological activity due to spatial chemical differences. A possible explanation for the increased cardiotoxicity associated with R (−) configuration is that cardiac sodium channels may be stereo-selectively beneficial in the uptake of local anesthetics. Scientists were able to generate local anesthetics as a single enantiomer commercially thanks to improvements in extraction methods and stereo-selective synthesis. As a result, ropivacaine was created, which at the time of its marketing included more than 99% S (−) form (RopivacaineSimpson et al., 2005).

### 1.4 Metabolism and elimination

Ninety-four percent of ropivacaine is linked to plasma proteins, primarily to 1-acid glycoprotein (Simpson et al., 2005). The metabolization occurs in the liver, mainly by cytochrome P450 (CYP) 1A2, to 3-hydroxi-ropivacaine (3-OH-ropivacaine) and by CYP3A4 by N-dealkylation to produce 2′,6′-pipecoloxylidide. The minor metabolites are 4-OH-ropivacaine and 2-OH-methyl ropivacaine are generated (Ekstrom and Gunnarsson, 1996; Lee et al., 1989). Eighty-six percent of the drug’s excretion following a single intravenous dose administration is accounted for by the kidney, which is the primary excretory organ for ropivacaine. The total dose, the method of administration, the patient’s hemodynamic and circulatory state, and the vascularity of the administration site all affect the plasma concentration of ropivacaine (Simpson et al., 2005).

### 1.5 Particularities

In animals and healthy individuals, ropivacaine has a substantially greater threshold for cardiotoxicity and central nervous system toxicity (Knudsen et al., 1997; Riff et al., 2022) than bupivacaine due to its lower lipophilicity and its stereoselective characteristics (Graf et al., 2002b; Dony et al., 2000). Another important characteristic of ropivacaine is its antibacterial activity *in vitro*, leading to the inhibition of the growth of *Staphylococcus aureus* (Bátai et al., 2002; Kampe et al., 2003), *Escherichia coli* (Bátai et al., 2002), and *Pseudomonas aeruginosa* (Riff et al., 2022).

The scientific literature includes a wide variety of pharmacological studies conducted on ropivacaine, using animal and human biological fluids and samples. These studies are important to describe the absorption, distribution, metabolization, and elimination of the drug. The most commonly used samples are plasma, either of human or animal origin (Mathieu et al., 2006), samples from tissue matrices are exclusively of animal origin, and the most frequently used is brain tissue (Kau et al., 2001). The most used analytical methods are High-Performance Liquid Chromatography with ultraviolet detection (HPLC-UV) and Liquid Chromatography coupled with Mass Spectrometry (LC-MS/MS) (Gaudreault et al., 2009). However, there are no articles describing ropivacaine quantification from tissues such as the lung, liver, heart, kidney, and no pharmacokinetic model is available.

The study’s goal was to propose a simple, inexpensive, adaptable, and reliable analytical approach with performance parameters validated per current guidelines in bioanalysis. It is a suitable method for high-throughput bioavailability, biomonitoring, bioequivalence, or other types of clinical studies.

## 2 Materials and methods

### 2.1 Reagents

Ropivacaine hydrochloride hydrate standard was acquired from Cayman Chemical (United States), Ropivacaine-d7 hydrochloride (deuterated internal standard - IS) and 3-OH-ropivacaine were purchased from Toronto Research Chemicals (Canada). HPLC grade acetonitrile and formic acid were acquired from VWR International (United States). Ultrapure water was produced by a Millipore Direct-Q3 (United States) system. Blank human plasma was provided by the local Transfusion Center from Targu Mures (Romania), with K3-EDTA used as an anticoagulant.

### 2.2 Apparatus and equipment

An LC-MS/MS system composed of a Perkin Elmer (United States) FX-10 liquid chromatograph coupled with an AB Sciex (United States) Triple TOF 4600 type mass spectrometer was used for chromatographic separation and quantification. Auxiliary equipment utilized: Eppendorf (Germany) 5430R centrifuge; Radwag (Poland) XA 52.3Y analytical scale; Velp Scientifica (Italy) ZX4 vortex mixer; JP Selecta (Spain) Ultrasons H-D ultrasonic bath; Eppendorf Research Plus (Germany) pipettes; Thermo (United States) Speed Vac sample evaporator and concentrator.

### 2.3 LC-MS/MS parameters

Chromatographic separation of ropivacaine, 3-OH-ropivacaine and ropivacaine-d7 (IS) was performed using a Gemini NX-18, 3.0 × 100 mm (3 μm particles) column (Phenomenex Inc.), thermostatted at 15°C, with a mobile phase consisting of 0.05%(V/V) formic acid in water (mobile phase A) and acetonitrile (mobile phase B) in gradient elution. The mobile phase gradient was as follows: 0–2-min 95% mobile phase A, 2–4 min 70% mobile phase A, 4–7 min 95% mobile phase A. The flow rate was constant at 0.4 mL/min. The injection volume was 1 μL for each sample. Detection was conducted in multiple reaction monitoring (MRM) mode for ropivacaine and 3-OH-ropivacaine, monitoring transition m/z 126.1 derived from m/z 275.1 ion at a collision energy of 19 V for ropivacaine, and m/z 126.1 derived from m/z 291.1 ion at a collision energy of 20 V for 3-OH-ropivacaine. For ropivacaine-d7 (IS) fragment m/z 133.1 derived from m/z 282.1 was monitored. Ionization of samples was performed using an electrospray ion source in positive mode, using the following ionization parameters: spray voltage 5000 V, vaporizer temperature 350°C, source gas1 pressure 35 psi, source gas2 pressure 30 psi, curtain gas pressure 25 psi. The total run-time of the method, including the column re-equilibration step, was 7 min per sample.

### 2.4 Standard solutions and analyte extraction

Stock solutions of ropivacaine and 3-OH-ropivacaine were prepared having concentrations of 500 μg/mL for ropivacaine and 100 μg/mL for the metabolite, respectively. These stock solutions were used to obtain the standard solutions in plasma for calibration curves, with a concentration range between 0.5 and 1,000 ng/mL for ropivacaine, and 1–1,000 ng/mL for 3-OH-ropivacaine. An IS solution having a concentration of 0.5 μg/mL of ropivacaine-d7 in acetonitrile was prepared. Quality control (QC) samples in plasma for ropivacaine with concentrations of 2 ng/mL, 30 ng/mL, 500 ng/mL, and 750 ng/mL, and with concentrations of 3 ng/mL, 30 ng/mL, 400 ng/mL, and 750 ng/mL for 3-OH-ropivacaine respectively, were prepared. 200 μL of the plasma standard and QC solutions were spiked with 100 μL IS solution and deproteinized by protein precipitation using 500 μL acetonitrile. After vortexation for 2 min and centrifugation for 3 min at 10,000 rpm supernatants were transferred to chromatographic vials and inserted into the autosampler for injection into the LC-MS system. The concentration of the internal standard (ropivacaine-d7) in the final sample solutions was 62.5 ng/mL.

### 2.5 Plasma sample preparation

For the analysis of plasma samples, a volume of 200 μL of plasma was spiked with 100 μL internal standard solution and deproteinized similarly to calibration standard and quality control solutions with 500 μL acetonitrile, vortexed for 2 min, centrifuged for 3 min at 10,000 rpm, with the supernatant being transferred to chromatographic vials and inserted into the auto-sampler and injected into the LC-MS system.

### 2.6 Method validation

Validation of the selectivity and sensitivity of the method was performed by comparing chromatograms of plasma samples spiked with the analytes and internal standard with blank plasma sample chromatograms. Six different blank matrix samples were evaluated for interfering peaks compared to samples spiked at the lower limit of quantification. The selectivity was calculated as the relative ratio between the peak area difference in blank and at the lower limit of quantification (LLOQ), *versus* analyte peak area at LLOQ.

Carry-over of the method was validated by injecting a blank sample after the highest calibration standard and evaluating if any interfering peaks appear at the retention time of the analytes or the internal standard. Carry-over was expressed as relative area ratio between the peak area in carry-over blank solutions *versus* standard solutions at LLOQ.

Concentrations were calculated by the instrument data system using the internal standard method. Calibration curves were linear and constructed from single calibration standards, using 1/y^2^ weighting factor. Linearity was determined based on the coefficient of correlation (R).

Intra-run and inter-run accuracy (expressed as relative difference between obtained and theoretical concentration, bias %) and precision (expressed as coefficient of variation, CV%) were determined by analyzing five replicates of LLOQ and each quality control sample, respectively, in the same analytical run and in different analytical runs.

The matrix effect was studied by analyzing four of each QC sample prepared in plasma, and one of each QC sample prepared in ultra-purified water. The recovery of the analyte and IS was determined by calculating the ratio of peak areas in the presence of plasma and in the absence of plasma. In order to determine the matrix effect, the matrix factor (ratio between area in the presence of matrix and in the absence of matrix, MF) of both the analyte and the IS was calculated, and then the CV% of the IS normalized matrix factor (MF of the analyte divided by the MF of the IS) was calculated for the four lower QC and four high QC samples prepared in plasma.

### 2.7 Anesthesia using plane block

**FIGURE 1:**
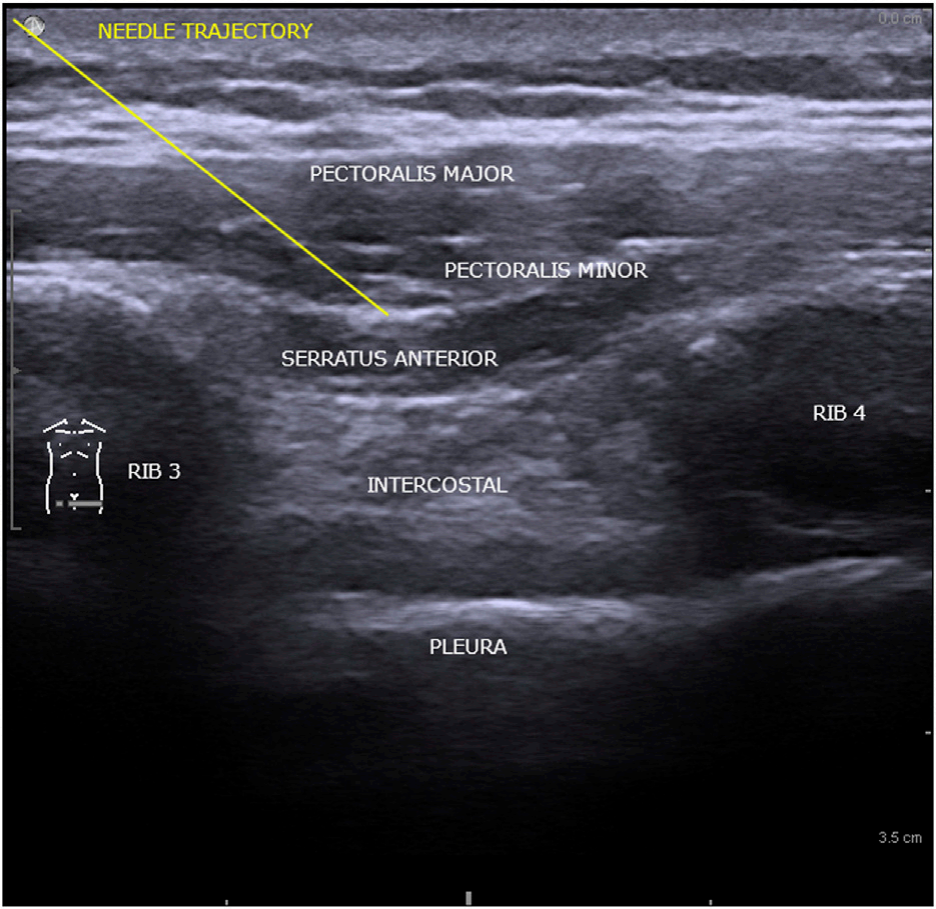
Ultrasonographic structures comprised in interpectoral and pectoserratus plane block.

Ethics Committee approval was obtained (1,515 from 09 December 2021). Interpectoral and pectoserratus plane block was used to provide anesthesia and analgesia for cardiac resynchronization therapy. The regional anesthetic technique required two administrations of a mixture in equal parts of ropivacaine 0.5% with lidocaine 1%, 2 mL/kg body weight, initially in the interfascial plane of the major and minor pectoral muscle, and a second between the minor pectoral and serratus anterior muscle. The procedure was carried out under ultrasound guidance, General Electric Vivid I6 sonograph was used, with an 8L probe, using a 50 mm 20G plex needle (Pajunk sonoplex). Adjusted doses of Midazolam were administered in association with regional anesthesia (Figure 1).

Blood samples from one patient were collected. The first sample was collected before plane block administration, and the other four after, at 1 hour, 3 hours, 6 hours, and 24 h after anesthetic block. A total of five blood samples were collected in vacutainers with clot activator and gel separator. Samples were then processed using a centrifuge, at 3,200 rpm, and the resulting plasma was stored in Eppendorf tubes at—80°C, until further utilization.

## 3 Results

### 3.1 Development and validation of the LC-MS/MS method

The method developed was validated following the EMA (European Medicines Agency) (Guideline on bioanalytical method validation, 2011) and FDA (Food and Drug Administration, United States) (Bioanalytical Method Validation Guidance for Industry, 2018) guidelines on bioanalytical method validation for the relevant parameters.

Under optimal mass spectrometric parameters and chromatographic conditions, the ropivacaine and its metabolite were detected in MRM mode by monitoring the same, common fragment ion m/z 126.1, but derived from the protonated parent pseudo molecular ion of each analyte, m/z 275.1 for ropivacaine and m/z 291.1 for 3-OH-ropivacaine, respectively. Due to the structural relationship between the ropivacaine and its metabolite, the mobile phase gradient was optimized in order to separate both analytes, without increasing the run-time too much. Retention times were 4.65 min for 3-OH-ropivacaine and 5.0 min for ropivacaine. Isotopically labeled ropivacaine-d7, used as an internal standard, was monitored through the selective fragmentation of parent ion m/z 282.1 to fragment m/z 133.1.

#### 3.1.1 Selectivity and sensitivity

**FIGURE 2:**
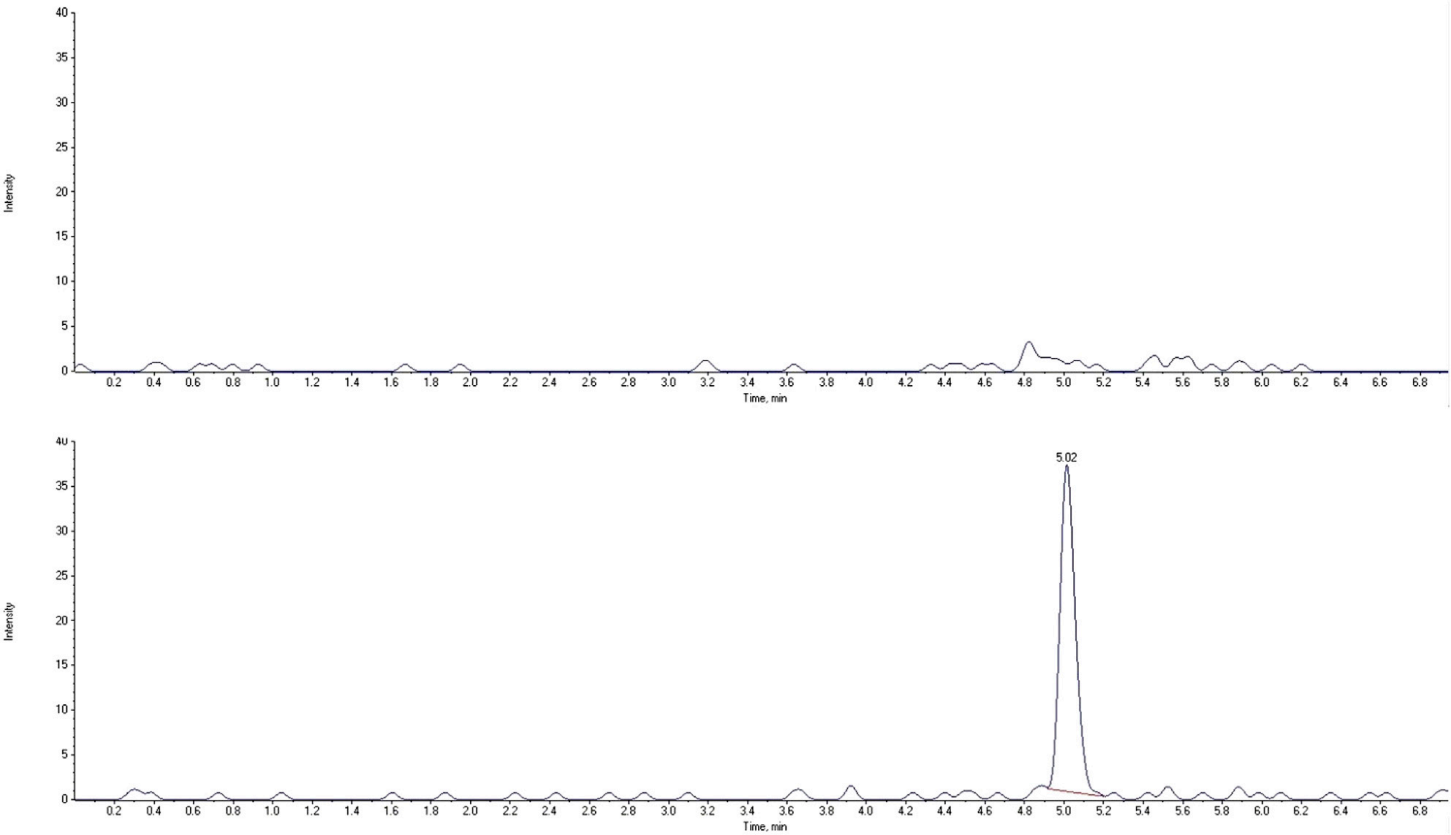
Extracted chromatograms of human blank plasma (top) and standard solution at the lower limit of quantification (bottom) for ropivacaine.

**FIGURE 3:**
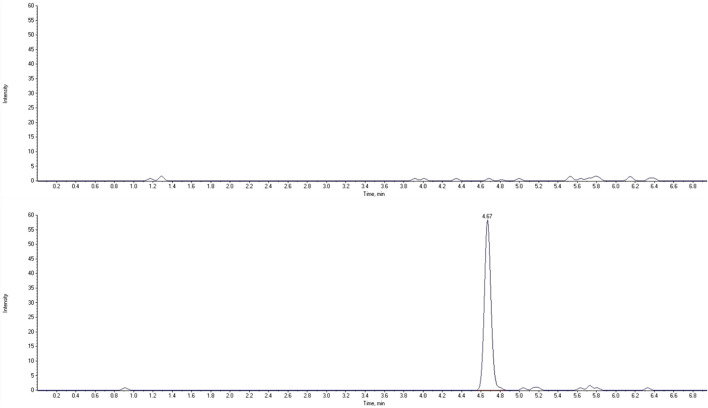
Extracted chromatograms of human blank plasma (top) and standard solution at the lower limit of quantification (bottom) for 3-OH-ropivacaine.

The LC-MS/MS method needs to provide selectivity between the analytes, IS and endogenous compounds from the biological matrix or other compounds (co-medication) which may be present in biological samples. For all five different blank samples, no interfering peaks with area greater than 20% of analytes peak area at the lower limit of quantification (LLOQ) were detected (Figures 2, 3). Results for selectivity validation are presented in Table 1.

**TABLE 1:** Selectivity with regards to ropivacaine and 3-OH-ropivacaine (n = 5).

*Ropivacaine series*		*3-OH-Ropivacaine series*	
Analyte	Average selectivity (±SD)	Analyte	Average selectivity (±SD)
Ropivacaine	94.21 (±6.03)	3-OH-Ropivacaine	98.89 (±2.48)
Ropivacaine-d7	99.92 (±0.17)	Ropivacaine-d7	99.89 (±0.23)

Legend: SD, Standard Deviation.

#### 3.1.2 Carry-over

The carry-over effect (contamination from one sample to the next) was studied by injecting a blank solution immediately after the most concentrated standard solution (1,000 ng/mL of each analyte) in each run of the validation. Areas in the blank samples appearing due to carry-over contamination were below 20% of LLOQ areas for analytes and below 5% of IS area (Table 2).

**TABLE 2:** Carry-over of ropivacaine and 3-OH-ropivacaine (n = 5).

*Ropivacaine series*		*3-OH-Ropivacaine series*	
Analyte	Average carry-over (±SD)	Analyte	Average carry-over (±SD)
Ropivacaine	0.00 (±0.00)	3-OH-Ropivacaine	4.82 (±3.91)
Ropivacaine-d7	0.13 (±0.30)	Ropivacaine-d7	0.30 (±0.45)

Legend: SD, Standard Deviation.

#### 3.1.3 Linearity of calibration curves and lower limit of quantification

Calibration curves were linear over the proposed concentration range with a lower limit of quantification (LLOQ) of 0.5 ng/mL for ropivacaine and 1 ng/mL for 3-OH-ropivacaine. The accuracy of recalculated concentrations of calibration standards was within acceptance limits for all calibration curves for each of the analytes, with no single calibration curve being compiled of less than 8 calibration standards which have passed acceptance criteria, and no more than 2 calibrations standards needed to be eliminated from any calibration curve, out of the total of 10 calibration levels. The coefficient of correlation (R) for all calibration curves was larger than 0.99, the acceptable limit to assess a linear correlation. The lower and upper limits of quantification were selected to assure the usability of the method for a wide array of studies and cover the variable concentration range resulting from the mode of administration of the anesthetic and the interindividual metabolic differences of human subjects, allowing for use in other types of studies as well. Five standard solutions at LLOQ of each analyte are analyzed to determine within and between run accuracy and precision at this level of concentration.

#### 3.1.4 Accuracy and precision

The accuracy and precision of the method assure the integrity and validity of the results. The LC-MS/MS method showed accuracy and precision within the ±15% acceptance limit for each analyte, both within as well as between runs (Tables 3–6). For the lower limit of quantification, the accuracy and precision were within the accepted ±20% acceptance limit for both ropivacaine and 3-OH-ropivacaine, for intra-run and inter-run testing.

**TABLE 3:** Overall intra-run accuracy and precision for ropivacaine (n = 5).

Nominal concentration ng/mL	Mean measured concentration ng/mL (±SD)	Precision (CV%)	Accuracy (bias%)
0.5	0.50 (**±**0.04)	8.0	0.1
2	2.16 (**±**0.09)	4.3	8.2
30	28.49 (±2.14)	7.5	−5.1
500	546.47 (±9.92)	1.8	9.3
750	711.67 (±70.45)	9.9	−5.1

Legend: SD, Standard Deviation; CV, Coefficient of Variation.

**TABLE 4:** Overall intra-run accuracy and precision for 3-OH-ropivacaine (n = 5).

Nominal concentration ng/mL	Mean measured concentration ng/mL (±SD)	Precision (CV%)	Accuracy (bias%)
1	1.00 (**±**0.07)	7.0	0.1
3	3.11 (**±**0.28)	8.9	3.7
30	31.11 (**±**2.21)	7.1	3.7
400	419.59 (**±**40.30)	9.6	4.9
750	772.30 (**±**61.67)	8.0	3.0

Legend: SD, Standard Deviation; CV, Coefficient of Variation.

**TABLE 5:** Overall inter-run accuracy and precision for ropivacaine (n = 5).

Nominal concentration ng/mL	Mean measured concentration ng/mL (±SD)	Precision (CV%)	Accuracy (bias%)
0.5	0.52 (**±**0.05)	9.7	3.0
2	2.08 (**±**0.18)	8.4	4.0
30	27.44 (**±**2.18)	7.9	−8.5
500	499.81 (**±**64.82)	13.0	0.0
750	783.45 (**±**77.66)	9.9	4.5

Legend: SD, Standard Deviation; CV, Coefficient of Variation.

**TABLE 6:** Overall inter-run accuracy and precision for 3-OH-ropivacaine (n = 5).

Nominal concentration ng/mL	Mean measured concentration ng/mL (±SD)	Precision (CV%)	Accuracy (bias%)
1	1.03 (**±**0.09)	8.7	3.2
3	3.03 (**±**0.30)	10.0	1.0
30	30.73 (**±**2.96)	9.6	2.4
400	408.20 (**±**51.92)	12.7	2.1
750	751.29 (**±**57.29)	7.6	0.2

Legend: SD, Standard Deviation; CV, Coefficient of Variation.

#### 3.1.5 Matrix effect

The internal standard (IS) normalized matrix effect (expressed as matrix factor, MF), calculated as the ratio of matrix factors of the analytes and the MF of the internal standard, and the coefficient of variation for IS normalized matrix factors proved that there is no significant effect of matrix on the areas of analyte peaks, and thus on results (Table 7).

**TABLE 7:** Matrix effect for ropivacaine and 3-OH-ropivacaine (n = 4).

*Ropivacaine series*			*3-OH-Ropivacaine series*		
Nominal concentration ng/mL	Average normalized MF	CV (%)	Nominal concentration ng/mL	Average normalized MF	CV (%)
2	1.006	0.703	3	1.028	11.383
30	0.895	6.294	30	0.923	4.025
500	1.074	6.092	400	0.972	6.648
750	1.052	9.639	750	1.030	6.954

Legend: MF, Matrix Factor; CV, coefficient of variation.

### 3.2 Analysis of patient plasma samples

**FIGURE 4:**
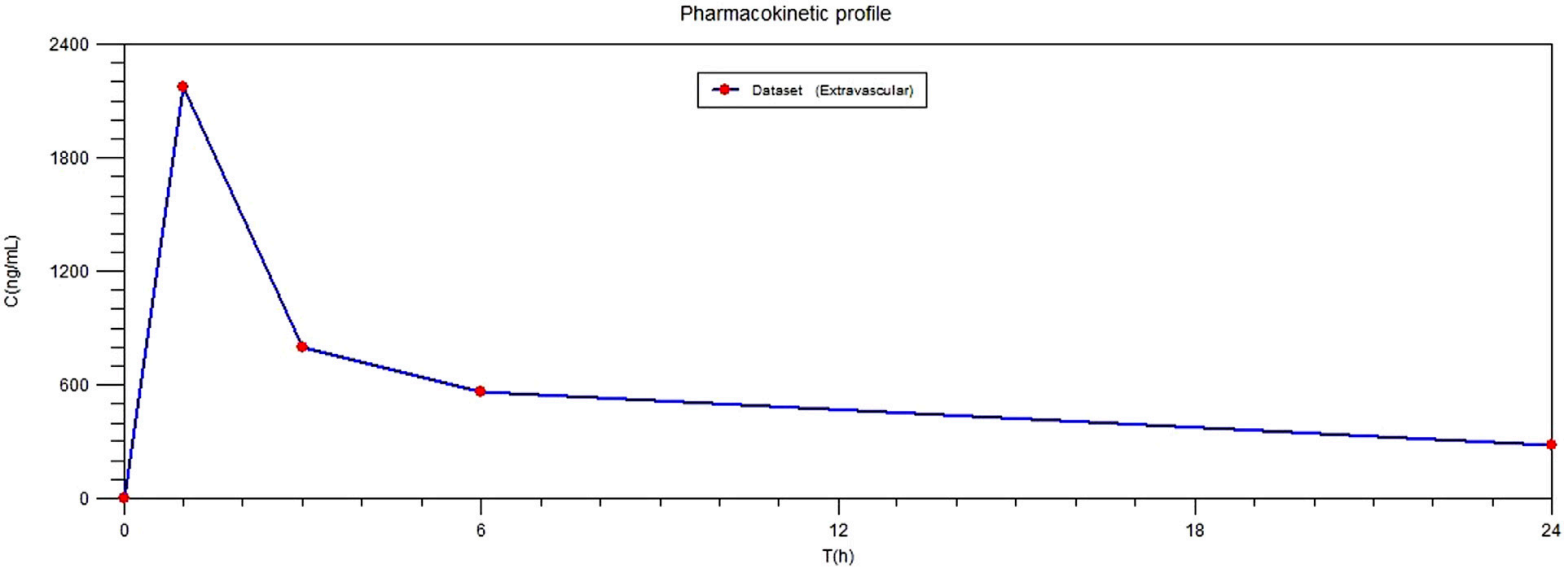
Plasmatic profile of ropivacaine in patient after plane block anesthesia.

As a model of applicability for the quantification method, plasma samples were collected from ore patient after plane block anesthesia. Samples were processed on the day of analysis and analyzed together with a calibration curve for ropivacaine and 3-OH-ropivacaine, a blank sample and QC samples for each analyte. Results show a quick plasmatic absorption of ropivacaine after the anesthesia, with a maximum plasmatic concentration of ropivacaine being reached 1 h after administration, followed by a slow but steady metabolization and elimination of ropivacaine (Figure 4). Concentrations of the metabolite are considerably lower and fluctuate at an approximately steady rate for the first 12 h after administration.

## 4 Discussions

Regional anesthetic techniques have gained a lot of popularity lately, especially due to the reduced adverse effects they exert on patients. However, cases of anesthetic toxicity are not non-existent, and when they do happen, their severity is not ignorable. Knowledge of pharmacokinetic details is important to predict unpleasant situations or avoid them altogether.

The practical advantages of ropivacaine over other local anesthetics are attributable to the fact that it is a pure S (−) enantiomer with moderate lipid solubility and high pKa. These physicochemical properties give it a high safety profile, combining the advantages of a latency time, intensity, and quality of a block like those of racemic bupivacaine and levobupivacaine, but with a lower toxicological tropism on the central nervous system and myocardium (Leone et al., 2008; Zink and Graf, 2008).

In most cases, toxicity after administration of local anesthetics occurs through intravenous infiltration. The systems most prone to toxicity are the central nervous system (CNS) and cardiovascular system. CNS toxicity is characterized by non-specific signs in the beginning such as shivering, muscle twitching, metallic taste in mouth, perioral paresthesia, up to tonic-clonic convulsions followed by hypoventilation, coma, and irreversible cardiovascular arrest. Cardiovascular system toxicity is characterized by tachycardia and initial hypertension followed by myocardial depression, arrhythmias, and total cardiovascular collapse (Zink and Graf, 2008). However, there is scarce data on systemic exposure to ropivacaine after administration of correctly calculated doses in the therapeutic range, although cases of Local Anesthetic Systemic Toxicity (LAST) with the production of convulsions and apnea in ultrasound-guided combined lumbar plexus and sciatic nerve block have recently been described in the literature (Aditya et al., 2021). The first line of treatment for LAST consists of rapid administration of a lipidic emulsion, circulatory support, seizure suppression therapy, and airway management (Guy, 2010).

According to The Toxin and Toxin Target Database (T3DB) (toxins, 2023), most of the ropivacaine pharmacokinetics is unknown except for drug metabolization and elimination. Moreover, there are no available data about the pathways of the drug, well as the tissue locations and toxicologic data (lethal dose, the toxic values, or the minimum risk level).

Only a few pharmacokinetic data are available regarding maximum concentration (Cmax) and time to peak plasma (Tmax) after administration of ropivacaine in lumbar-plexus and sciatic-nerve blocks using a GC-MS method in small samples (12 patients) (Vanterpool et al., 2006).

Although scarce, such data are important as they fill some gaps in the literature and provide us with plasma levels within the safety margin. The identification of plasma levels following accidents of the LAST type could serve to introduce, in addition to the maximum allowed dose, a cut-off value of the plasma concentration and to prevent toxicity through therapeutic drug monitoring.

Ropivacaine bioanalytical methods can assess its concentration in several types of samples such as biological fluids (plasma, cerebrospinal fluid, or urine) and tissue matrices (heart, brain, lung, liver, kidney, fatty tissue, muscle), not only for clinical biomonitoring purpose, but also in forensic medicine.

Further research is needed to fully understand the pharmacokinetics and pharmacodynamics of local anesthetics.

As a result of the above-mentioned facts, a question arises naturally: what would be the strengths of our LC-MS/MS method for ropivacaine dosage from biological matrices such as plasma.

- a validated, precise method for determining the plasma concentration of ropivacaine, with remarkably high specificity, without the interference of endogenous compounds or concomitant treatments.
- the possibility of using the method for therapeutic drug monitoring.

There are several methods published in scientific literature which describe the use of different types of analytical methodologies for biomonitoring, pharmacokinetic or forensic purposes (Mathieu et al., 2006; Sawaki et al., 2009; Tonooka et al., 2016; Zhang et al., 2019; Lamy et al., 2020; Cui et al., 2023). In a recent publication, Cui et al. (Cui et al., 2023) describe an LC-MS method for quantification of ropivacaine in pig plasma which is very simple and quick, allowing for high-throughput analysis, however, the lack of an internal standard increases the possibility of erroneous measurements due to different types of systematic laboratory errors which can occur during sample preparation and due to technical limitations of the equipment. Sawaki et al. (Sawaki et al., 2009) had a similar approach when measuring ropivacaine levels from rabbit plasma, also lacking internal standard and using protein precipitation, which although generally considered to be one of the most highly reproducible pre-analysis sample preparation methods, when not using an internal standard can still lead to random sample preparation errors which will not be corrected. Mathieu et al. (Mathieu et al., 2006) also used protein precipitation, while also using etidocaine as an internal standard. This approach increases the reproducibility of the measurements and results; however, it necessitates additional development steps for the LC-MS methodology. In comparison, using an isotopic internal standard assures the method described in this manuscript is less susceptible to systematic errors without adding extra steps during chromatographic and mass spectrometric method development, or sample preparation, as the internal standard will elute, be detected, and can be processed under the same conditions as the analyte.

Another approach for ropivacaine analysis described in the literature is the one using more complex sample preparation and clean-up methods, such as Lamy et al. (Lamy et al., 2020) using sample dialysis on human plasma samples, or Tonooka et al. (Tonooka et al., 2016) using solid-phase extraction for human serum samples. Both these methods add costs to the analysis and increase sample analysis time, reducing the throughput and feasibility. At the same time, many of the previously published methods have longer run-times per sample (Mathieu et al., 2006; Sawaki et al., 2009; Tonooka et al., 2016; Lamy et al., 2020) which can be a disadvantage when doing measurements that need high-throughput (bioavailability studies) or short turn-around times for the analysis results (forensics, toxicology, biomonitoring or dose adjustment).

We consider that the work brings the first concrete data regarding the level of plasma concentrations and the underlying systemic exposure following intra-fascial administration. The determined plasma levels can be constituted as therapeutic intervals in which the mentioned local anesthesia proceeds under safe conditions for patients. The use of the echo-guided technique led to a decrease in administered doses. Plasma concentration measurements can serve as safety benchmarks of systemic exposure in case of LAST accidents that cannot be explained by erroneous administration techniques or inappropriate doses, but by individual reactivity and particular pharmacokinetics.

## Data Availability

The raw data supporting the conclusion of this article will be made available by the authors, without undue reservation.
