# Supplementary material for: LC-MS/MS assisted biomonitoring of ropivacaine and 3-OH-ropivacaine after plane block anesthesia for cardiac device implantation
Source: Front Mol Biosci. 2023 Sep 27;10:1243103. doi: 10.3389/fmolb.2023.1243103 (PMC10566374; doi:10.3389/fmolb.2023.1243103)
Supplement: Supplementary file 1 [file Table1.doc]

Tables

Table 1. Selectivity with regards to ropivacaine and 3-OH-ropivacaine (n = 5)

| ***Ropivacaine series*** | | ***3-OH-Ropivacaine series*** | |
| --- | --- | --- | --- |
| **Analyte** | **Average selectivity**  **(± SD)** | **Analyte** | **Average selectivity**  **(±SD)** |
| Ropivacaine | 94.21 (±6.03) | 3-OH-Ropivacaine | 98.89 (±2.48) |
| Ropivacaine-d7 | 99.92 (±0.17) | Ropivacaine-d7 | 99.89 (±0.23) |

*Legend: SD – Standard Deviation*

Table 2. Carry-over of ropivacaine and 3-OH-ropivacaine (n = 5)

| ***Ropivacaine series*** | | ***3-OH-Ropivacaine series*** | |
| --- | --- | --- | --- |
| **Analyte** | **Average carry-over**  **(±SD)** | **Analyte** | **Average carry-over**  **(±SD)** |
| Ropivacaine | 0.00 (±0.00) | 3-OH-Ropivacaine | 4.82 (±3.91) |
| Ropivacaine-d7 | 0.13 (±0.30) | Ropivacaine-d7 | 0.30 (±0.45) |

*Legend: SD – Standard Deviation*

Table 3. Overall intra-run accuracy and precision for ropivacaine (n=5)

| **Nominal concentration ng/mL** | **Mean measured concentration ng/mL (±SD)** | **Precision (CV%)** | **Accuracy (Bias%)** |
| --- | --- | --- | --- |
| **0.5** | 0.50 (**±**0.04) | 8.0 | 0.1 |
| **2** | 2.16 (**±**0.09) | 4.3 | 8.2 |
| **30** | 28.49 (±2.14) | 7.5 | -5.1 |
| **500** | 546.47 (±9.92) | 1.8 | 9.3 |
| **750** | 711.67 (±70.45) | 9.9 | -5.1 |

*Legend: SD – Standard Deviation; CV - Coefficient of Variation*

Table 4. Overall intra-run accuracy and precision for 3-OH-ropivacaine (n=5)

| **Nominal concentration ng/mL** | **Mean measured concentration ng/mL (±SD)** | **Precision (CV%)** | **Accuracy (Bias%)** |
| --- | --- | --- | --- |
| **1** | 1.00 (**±**0.07) | 7.0 | 0.1 |
| **3** | 3.11 (**±**0.28) | 8.9 | 3.7 |
| **30** | 31.11 (**±**2.21) | 7.1 | 3.7 |
| **400** | 419.59 (**±**40.30) | 9.6 | 4.9 |
| **750** | 772.30 (**±**61.67) | 8.0 | 3.0 |

*Legend: SD – Standard Deviation; CV - Coefficient of Variation*

Table 5. Overall inter-run accuracy and precision for ropivacaine (n=5)

| **Nominal concentration ng/mL** | **Mean measured concentration ng/mL (±SD)** | **Precision (CV%)** | **Accuracy (Bias%)** |
| --- | --- | --- | --- |
| **0.5** | 0.52 (**±**0.05) | 9.7 | 3.0 |
| **2** | 2.08 (**±**0.18) | 8.4 | 4.0 |
| **30** | 27.44 (**±**2.18) | 7.9 | -8.5 |
| **500** | 499.81 (**±**64.82) | 13.0 | 0.0 |
| **750** | 783.45 (**±**77.66) | 9.9 | 4.5 |

*Legend: SD – Standard Deviation; CV - Coefficient of Variation*

Table 6. Overall inter-run accuracy and precision for 3-OH-ropivacaine (n=5)

| **Nominal concentration ng/mL** | **Mean measured concentration ng/mL**  **(±SD)** | **Precision (CV%)** | **Accuracy (Bias%)** |
| --- | --- | --- | --- |
| **1** | 1.03 (**±**0.09) | 8.7 | 3.2 |
| **3** | 3.03 (**±**0.30) | 10.0 | 1.0 |
| **30** | 30.73 (**±**2.96) | 9.6 | 2.4 |
| **400** | 408.20 (**±**51.92) | 12.7 | 2.1 |
| **750** | 751.29 (**±**57.29) | 7.6 | 0.2 |

*Legend: SD – Standard Deviation; CV - Coefficient of Variation*

Table 7. Matrix effect for ropivacaine and 3-OH-ropivacaine (n = 4)

| ***Ropivacaine series*** | | | ***3-OH-Ropivacaine series*** | | |
| --- | --- | --- | --- | --- | --- |
| **Nominal concentration ng/mL** | **Average normalized MF** | **CV (%)** | **Nominal concentration ng/mL** | **Average normalized MF** | **CV (%)** |
| 2 | 1.006 | 0.703 | 3 | 1.028 | 11.383 |
| 30 | 0.895 | 6.294 | 30 | 0.923 | 4.025 |
| 500 | 1.074 | 6.092 | 400 | 0.972 | 6.648 |
| 750 | 1.052 | 9.639 | 750 | 1.030 | 6.954 |

*Legend: MF – Matrix Factor; CV - coefficient of variation*
